# Comprehensive DNA Signature Discovery and Validation

**DOI:** 10.1371/journal.pcbi.0030098

**Published:** 2007-05-18

**Authors:** Adam M Phillippy, Jacquline A Mason, Kunmi Ayanbule, Daniel D Sommer, Elisa Taviani, Anwar Huq, Rita R Colwell, Ivor T Knight, Steven L Salzberg

**Affiliations:** 1 Center for Bioinformatics and Computational Biology, University of Maryland, College Park, Maryland, United States of America; 2 Canon U.S. Life Sciences, Rockville, Maryland, United States of America; 3 Center of Marine Biotechnology, University of Maryland Biotechnology Institute, Baltimore, Maryland, United States of America

## Abstract

DNA signatures are nucleotide sequences that can be used to detect the presence of an organism and to distinguish that organism from all other species. Here we describe Insignia, a new, comprehensive system for the rapid identification of signatures in the genomes of bacteria and viruses. With the availability of hundreds of complete bacterial and viral genome sequences, it is now possible to use computational methods to identify signature sequences in all of these species, and to use these signatures as the basis for diagnostic assays to detect and genotype microbes in both environmental and clinical samples. The success of such assays critically depends on the methods used to identify signatures that properly differentiate between the target genomes and the sample background. We have used Insignia to compute accurate signatures for most bacterial genomes and made them available through our Web site. A sample of these signatures has been successfully tested on a set of 46 Vibrio cholerae strains, and the results indicate that the signatures are highly sensitive for detection as well as specific for discrimination between these strains and their near relatives. Our approach, whereby the entire genomic complement of organisms are compared to identify probe targets, is a promising method for diagnostic assay development, and it provides assay designers with the flexibility to choose probes from the most relevant genes or genomic regions. The Insignia system is freely accessible via a Web interface and has been released as open source software at: http://insignia.cbcb.umd.edu.

## Author Summary

Now that the genome sequences of hundreds of bacteria and viruses are known, we can design tests that will rapidly detect the presence of these species based solely on their DNA. Such tests have a wide range of applications, from diagnosing infections to detecting harmful microbes in a water supply. These tests can detect a pathogen in a complex mixture of organic material by recognizing short, distinguishing sequences—called DNA signatures—that occur in the pathogen and not in any other species. We present Insignia, a new computational system that identifies DNA signatures of any length in bacterial and viral genomes. Insignia uses highly efficient algorithms to compare sequenced bacterial and viral genomes against each other and to additional background genomes including plants, animals, and human. These comparisons are stored in a database and used to rapidly compute signatures for any particular target species. To maximize its utility for the community, we have made Insignia available as free, open-source software and as a Web application. We have also validated 50 Insignia-designed assays on a panel of 46 strains of *Vibrio cholerae,* and our results show that the signatures are both sensitive and specific.

## Introduction

Modern health and security concerns have raised interest in the real-time detection and identification of pathogenic microbes. Bacterial and viral pathogens have always represented one of the greatest threats to human health, and in recent times this threat increased due to the possibility of engineered biological agents. For these and other reasons, the genome sequencing field has targeted and sequenced the complete genomes of hundreds of bacteria and thousands of viruses over the past decade, with many more sequences expected to appear in the near future. These sequences now make it possible to develop probe-based assays capable of identifying any of hundreds of organisms in environmental and clinical samples. Such assays rely on detecting a DNA sequence that distinguishes the target organism from all other known bacteria and viruses and from background material, which could include DNA from humans, other animals, plants, or other species. A probe that accurately distinguishes between a target genome—or set of genomes—and all other background genomes is termed a signature sequence.

By our definition, a signature sequence must be conserved among a set of target genomes and dissimilar to any sequence in the surrounding environment. To detect a target with existing technology such as qPCR assays, signatures must be relatively short; however, if they are too short, they will not be unique. For example, because there are only 4^10^ ≈ 1 million 10-bp (base-pair) sequences, and a typical bacterial genome is more than 1 million bp in length, most 10-mers will be shared by many genomes and therefore make unsuitable signatures. Increasing the length, *k*, of the signature alleviates this problem, but if *k* is too large, it may not be possible to find a signature shared by a set of target genomes. Therefore, there is a tradeoff between signature sensitivity (the number of genomes that share the signature) and specificity (the number of genomes that do not possess the signature). For instance, a long signature may be highly specific to a particular strain or isolate, but it may not be sensitive enough to detect closely related strains that might cause the same disease or have other shared phenotypic characteristics. Because genomic sequence is nonrandom, and only a small sample of genomes has been sequenced, it is difficult to estimate an optimal signature length. In practice, signature length is usually determined by the constraints of the detection technology (e.g., ~20 bp for PCR primers).

Current probe-based technologies are generally based on either PCR or microarray hybridization. These methods are beginning to replace traditional gel-based fingerprinting because they can more effectively differentiate between closely related microbes [1]. Microarray methods are particularly promising because of their ability to multiplex many probes on a single chip [1–3], improving both the redundancy and capabilities of the diagnostic. PCR does not multiplex as nicely; however, it remains popular because of its robustness, speed, and low cost [4–6]. Unlike restriction fingerprinting, both PCR and microarray methods require explicit knowledge of the underlying DNA sequence, therefore necessitating probe design.

Traditional probe design strategies have focused on single genes or other loci that are determined a priori to be useful in distinguishing one target organism from another. Examples include genes that are associated with phylogenetic distance (e.g., 16S rRNA genes) and variable number tandem repeats (VNTRs). In the former case, where the gene or locus is conserved among target and nontarget organisms, gene sequence alignments would be used to aid in probe design. Probes would then be manually designed and screened for sensitivity and specificity to the target. Those assays failing to identify all target organisms, or producing false positives, would be invalidated and the design revised. This manual screening made diagnostic assay design expensive and only worth doing for a few select pathogens. Alternatively, variable number tandem repeats (VNTRs) have proven very useful in classifying and distinguishing many closely related strains of bacteria, such as Bacillus anthracis whose 16S rRNA sequences are identical [7,8]. Although these methods are effective, they only provide a limited number of signatures, which are not always sufficient to identify bacteria or viruses in a new sample; in particular, if the sample contains an unknown strain, it might contain genetic variability in precisely the region for which assays are designed. Thus, in general, one would like to have as many assays available as possible. Insignia addresses this by using the complete genome to generate *all* unique signatures, from which the assay designer can choose those that are best-suited for a particular application.

### Prior Work

Recent increases in the amount of available genomic sequence have made it possible to largely automate the design and screening of probes via computational search algorithms. Large-scale computational prediction of DNA signatures was first undertaken for the Biological Aerosol Sentry and Information System (BASIS), deployed at the Salt Lake City Olympic Games in 2002 [9,10]. The related BioWatch project operates by collecting and analyzing airborne microbial samples for known pathogens, using PCR probe-based detection methods. Newer aerosol detection systems, such as the Autonomous Pathogen Detection System (APDS) [11], automate the process, and can identify a known bioweapon in 0.5 to 1.5 hours [12]. Similar techniques are not limited to aerosols, and can be used in clinical or agricultural settings [13].

The success of these assays depends on both the available sequence databases and the computational methods used to identify signatures that differentiate the threat organisms from the background. Signature design for both BASIS and BioWatch was handled by Lawrence Livermore National Laboratories (LLNL), and what began as a simple proof-of-concept BLAST search at LLNL evolved into the sophisticated KPATH signature pipeline [14]. KPATH identifies sequences shared by a collection of target genomes, yet unique with respect to all other microbial genomes, and is notable for its ability to handle such a large search space. Other methods for probe selection more rigorously address hybridization efficiency (binding energy, self-hybridization, etc.), but do not scale well for large target and background sets [15–18]. Most notable are the approaches that promise the scalability of KPATH combined with the hybridization considerations of the other methods [19,20].

Because of its history of use in real-world diagnostic systems, a more detailed description of KPATH is warranted. It consists of four major components. First, a whole-genome multi-alignment is performed on a set of target genomes. This produces a “consensus gestalt,” which represents the sequences that are conserved in all the target genomes. Next, this consensus is matched against a database of background sequences using Vmatch [21]. This step computes all exact matches between the target consensus and the background. Matching sequences are masked out to create a “uniqueness gestalt,” which represents all sequences that are shared between target genomes and unique with respect to the background. Third, signature sequences are supplied to the Primer3 program [22], which designs PCR assays based on those sequences. Primer3 produces a set of oligos suitable for testing by a TaqMan PCR assay: a forward primer, a reverse primer, and an intervening probe oligomer [23]. Finally, assay candidates are screened using BLAST [24] for near matches that might disrupt the hybridization process, and ranked according to their satisfaction of PCR experimental constraints. The result of this four-stage process is a set of ranked, prescreened assays, which are then subjected to rigorous laboratory validation. The transition to these computational methods from previously manual design methods has resulted in greatly increased design efficiency by limiting the number of assays that fail during laboratory validation.

### Insignia

While highly innovative, the KPATH pipeline is not publicly available, and many of the sequences and signatures remain secret. In addition, KPATH requires significant computing resources (hours of computing time on a 24-CPU server [14]), which are beyond the means of many investigators. In contrast, Insignia is a transparent, highly accessible signature pipeline, with the entire system being controlled by a Web interface and all supporting software released under an open source model. Additionally, Insignia dramatically accelerates the discovery process by precomputing exact sequence matches for all genomes and storing this information in a specialized data structure for rapid retrieval.

Using the Insignia Web interface, users select a desired signature length and a set of target genomes. After query submission, the system analyzes the stored match information, and identifies signature candidates in less than one minute. Candidates may then be further screened using experimental constraints (melting temperature, GC content, etc.), or using further computational criteria, such as the existence of near matches that may cause cross-hybridization. The integrated Gemina database (http://gemina.tigr.org), which includes detailed annotation and supplementary epidemiological information for major pathogens, provides further support for signature selection. This rich metadata allows the formulation of complex queries such as “find signatures shared by all enteric Escherichia coli,” and it allows the user to search for signatures in the context of the surrounding annotation. Insignia can compute signatures for any microbial genome in GenBank (both draft and complete), and screens signatures against a comprehensive background including all bacterial, archaeal, and viral sequences, plus additional eukaryotic sequences from the National Center for Biotechnology Information (NCBI) RefSeq database [25].

## Results

Insignia was used to develop assays for the identification of V. cholerae at the species level using a TaqMan Real-Time qPCR format. The initial version of Insignia queried a database that was populated with ~300 bacterial genomes, including one strain of V. cholerae (O1 biovar El Tor strain N16961), and four near neighbors in the family *Vibrionaceae* (three *Vibrio* and one *Photobacterium* species). Thus the question for Insignia was: among all available DNA sequences, what sequences are unique to V. cholerae? The Insignia Web interface was used to retrieve all 20-mers unique to V. cholerae, from which 50 TaqMan assays were designed. A similar query with the current version of Insignia takes 10 s and returns 34,122 signatures of varying lengths.

**Figure 1:**
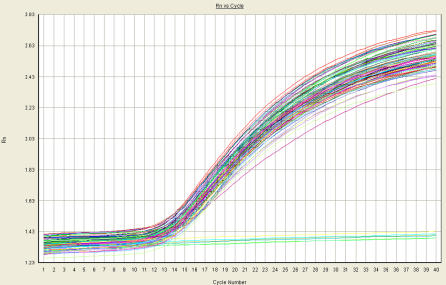
Inclusive TaqMan Assay Displaying Increased Fluorescence due to Target Amplification for All 46 V. cholerae Strains Tested, and No Fluorescent Activity among the E. coli Negative Controls Relative florescence intensity for 40 PCR cycles is shown.

**Figure 2:**
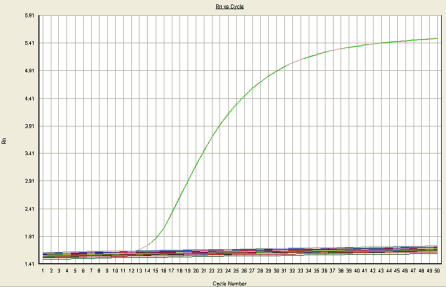
Exclusive TaqMan Assay Displaying Increased Fluorescent Activity for the Reference Strain of V. cholerae and No Fluorescent Activity among the 23 Non-Cholera Strains Relative florescence intensity for 50 PCR cycles is shown.

To test whether the signature assays were broadly inclusive of V. cholerae strains, the 50 assays were tested against a panel of 46 strains of V. cholerae comprising a global distribution of both clinical and environmental strains from all major serotypes. To test whether they excluded non-cholera vibrios, the assays were additionally tested against a panel of 22 nearest-neighbor species in the family *Vibrionaceae*, along with one E. coli control. Figures 1 and 2 show example inclusive and exclusive qPCR results, respectively.

**Figure 3:**
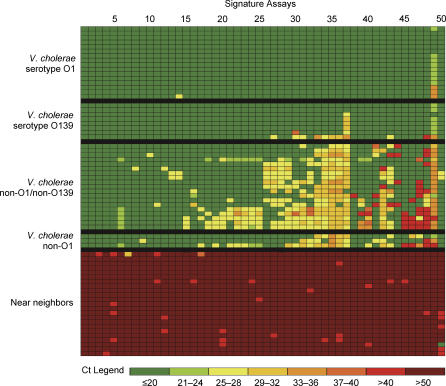
TaqMan Validation Results for the 50 Assay Designs Tested on 46 V. cholerae, 22 Near Neighbors, and One E. coli Control Organisms are grouped vertically, and assays are sorted horizontally by effectiveness. Each colored box represents the Ct value for one of the 3,450 validation experiments. For example, assays 1–5 show strong amplification for all V. cholerae strains and heavily delayed or failed amplification for all other organisms.

Figure 3 summarizes the validation results for the 50 assays, covering 69 organisms, and totaling 3,450 experiments. Each square in Figure 3 represents one experiment, with color indicating the qPCR Ct value (the number of PCR cycles before amplification is detected). Green and yellow squares indicate relatively rapid amplification while orange and red indicate delayed or failed amplification. (For a grayscale version of Figure 3, see Figure S1.) As Figure 3 makes clear, most assays detected all V. cholerae strains, with approximately half of the assays providing strong detection capability for every one of these diverse strains. The effectiveness of some assays deteriorated slightly for the non-O1/O139 serotypes, although they still provided positive results. This was to be expected, however, given that only a single V. cholerae strain (of serotype O1) was available to Insignia. Additional genomic sequences from the other serotypes would have undoubtedly removed many of these less-efficient signatures from the Insignia output. Gardner et al*.* explore this phenomenon further in the context of viral signature development [26].

In addition to successful detection of a wide variety of V. cholerae strains, all but one of the tested assays (98%) were able to successfully discriminate between V. cholerae and its near neighbors. Furthermore, 1,115 of the 1,150 exclusive tests (97%) had Ct values >50, indicating that all of the tested V. cholerae signatures are either absent or significantly divergent from the other members of *Vibrionaceae*.

Assay signature sequences are provided in Table S1, inclusive and exclusive strain information in Table S2, and detailed qPCR results for all 3,450 validation experiments in Table S3. This information is also available from the Insignia Web site.

## Discussion

Our validation results indicate that whole genome signature discovery, whereby the entire genomic complement of organisms are compared to identify probe targets, is a promising new tool for diagnostic assay development. This approach provides assay designers with the flexibility to choose probes of the proper length from the most relevant genes or genomic regions, while avoiding sequences known to contain no suitable signatures. Insignia also achieves unmatched scale by screening all microbial genomes in GenBank against a comprehensive background, while providing rapid access to DNA signatures through its Web interface.

Insignia outputs signature candidates, rather than high confidence, laboratory-validated signatures. However, our results demonstrate that most of these candidates can work quite well as laboratory assays. Due to the limited availability of genomic sequence in public databases (relative to the diversity of all organisms), and the possibility of near-match cross-hybridization, it is difficult to validate a genomic signature via purely computational methods. Instead, Insignia provides a computational screening regimen that eliminates many invalid signatures, so that laboratory validation may focus on the most likely candidates. Additional sequencing will help overcome the computational limitation, and future work on Insignia will be focused on screening signature candidates for near matches that may result in cross-hybridization.

In addition to the computational restrictions, limitations of TaqMan PCR have been demonstrated for rapidly diverging target genomes, such as hepatitis and HIV viruses [26,27]. However, for typical bacterial targets, TaqMan assays remain one of the most rapid and sensitive methods for signature detection. In the case where TaqMan is inadequate, different detection technologies, such as chip-hybridization methods, could be used to remove the TaqMan requirement for three adjacent probes and to provide greater signature redundancy. Insignia would easily support the design of such assays.

Viruses pose significant challenges for all detection methods because of their small genomes and high mutation rates. The Insignia database contains thousands of viral genomes; however, for large target sets there are often no conserved signatures. To address highly divergent targets, future Insignia versions may include the ability to identify signatures with degenerate bases, for cases where no exact signature is shared between them. An alternative is to compute the minimum signature set, where each signature might not identify every target, but the set contains at least one identifying signature for each target. This approach is particularly suited for chip assays where signatures can be multiplexed. A related approach selects combinations of non-unique probes, such that certain viral strains can be identified by their hybridization pattern [28]. Insignia support for specialized viral diagnostics is left for future work.

## Materials and Methods

Insignia provides real-time signature retrieval for an arbitrary set of target and background genomes. This requires the vast majority of computational work to be done in advance and cached, so that a minimum amount of computation is necessary at the time of the query. To accommodate this, Insignia is designed as two separate components: the match pipeline and the signature pipeline. This distinction separates the computationally intensive matching step from the much simpler signature generation step, and allows sequence matches to be recomputed offline as new genomes become available. While the matches may take days to compute, the signatures can be extracted from this cached information in seconds.

### Match pipeline.

The function of the match pipeline is to identify exact matches between all pairs of target and background sequences in the database. The size of the Insignia sequence database is currently about 60 billion nucleotides, and even with the linear-time algorithms described below, this is too large to search in real time. Some computational effort is saved by limiting targets to microbial genomes only, but the process of matching all pairs of target and background genomes remains expensive.

To complete the matching phase within a reasonable amount of time, all exact matches of 18 bp or longer are first identified using MUMmer [29–31], a linear time and space suffix tree matching algorithm. To expedite the process, MUMmer searches are partitioned across a 192-node Linux cluster. Even with the use of an efficient search algorithm, however, the size of the database and the high repeat content of many genomes cause the size of the output—the number of matches between all pairs of genomes—to reach unmanageable levels (e.g., the number of matches can be quadratic with respect to the size of the genomes). To combat this problem, matches are converted to a minimalized “match cover” data structure, described next. This structure saves substantial space and later provides a convenient mechanism for computing signatures.

**Figure 4:**
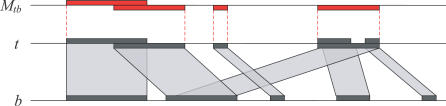
A Match Cover (*M_tb_*) Constructed from the Exact Matches between a Target (*t*) and Background (*b*) Genome *M_tb_* intervals (red boxes) represent regions of the target with a contiguous match to the background (gray boxes).

The *match cover*, *M_tb_*, of a target genome *t*, with respect to some background genome *b*, is simply the list of intervals on *t* that are covered by contiguous, exact matches to genome *b*. To eliminate redundancy, all intervals contained within larger intervals are removed, but overlapping intervals are not merged. This assures that every subinterval matches contiguously to some portion of the background sequence, and every maximal match to the background is contained by a single interval (Figure 4). After construction of the match cover, the intervals are sorted by their start position, and stored as a list of (start, length) pairs. Because this structure only stores the target “half” of the match data, space requirements are reduced by eliminating irrelevant background match coordinates. What remains is a minimal set of intervals on genome *t* that exactly match some part of genome *b*.

In addition to storing only the target half of the matches, the match cover eliminates redundant information caused by repetitive sequences. Take for instance, two potential target genomes *t* and *u*. Because all target genomes are, by default, part of the background, two match covers will be created, *M_tu_* and *M_ut_*. Now assume an identical repeat occurs *x* times in *t*, and *y* times in *u*. A list of exact matches (start *t*, start *u*, and length) would require 3*xy* integer values to represent the repeat, while the match covers would require only 2(*x + y*) combined values. Therefore, even when storing both halves of a match set (*t → u* and *u → t*), the match cover is more efficient in dealing with repeats. This behavior was empirically tested for an all-versus-all comparison of ~300 bacterial genomes, and the match cover reduced the match list from its original size of 78 GB to just 2 GB. This 39-fold space reduction demonstrates the prevalence of repetitive matches in real data and the utility of the match cover structure. Considering the match cover is simply a list of intervals, standard data compression could be applied to obtain further space savings.

The match cover is not a lossless conversion, however, because it discards information about where a match occurred in the background. The information is nonetheless sufficient for signature computation, where it suffices to know which regions of a target are unique. Furthermore, by excluding irrelevant background match positions, large background databases can be accommodated without drastically increasing the match cover size, and draft quality genomic sequences can be incorporated without difficulty. As the next section will show, the match cover encapsulates all the necessary information for signature discovery and allows for the rapid construction of signatures for any set of target and background genomes in linear time.

For perspective, it is worth mentioning that the match cover is an equivalent, interval representation of matching statistics [32,33]. Both formalizations represent the longest contiguous match beginning at any position of a sequence, but our interval representation is space-efficient and easier to interpret in the context of signature discovery. Rahmann also leverages the properties of matching statistics in describing a “jump list” for the discovery of DNA probes [20], and it is interesting to note that although the match cover and jump list were arrived at independently, they are analogous given their shared utilization of matching statistics.

### Signature pipeline.

The function of the signature pipeline is to generate valid signatures for any set of target and background genomes. Because there are thousands of possible targets and many more backgrounds, combinatorics rules out the pre-computation of all signatures; however, it is possible to generate signatures from the match information with minimal overhead. The pipeline for doing so is divided into two parallel stages, corresponding to the two primary criteria a valid signature must meet: 1) a signature must be shared by all genomes in the target set; and 2) a signature must not exist in any genome in the background set.

The first stage computes a list of *k*-mers (DNA sequences of length *k*) shared by the set of target genomes. This could be determined by computing a whole-genome multi-alignment among the targets; however, multi-alignment algorithms are too slow for a real-time application (e.g., 30 min to align three E. coli strains [34]). Alternatively, shared *k*-mers could be identified by intersecting *k*-mer tables for each target genome, but these tables would have to be constructed on the fly for each *k* (since *k* is specified by the user at run time), which would also be costly. Instead, Insignia utilizes the pre-computed match cover to quickly infer shared *k*-mers for any length *k* greater than the minimum match length (currently 18 bp) used to build the match covers.

**Figure 5:**
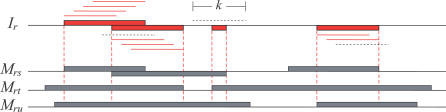
Shared *k*-mers (Red Lines) Obtained from an Intersection (*I_r_*) of Three Match Covers *M_rs_*, *M_rt_*, *M_ru_* *I_r_* intervals (red boxes) represent regions of the reference *r* shared with all other target genomes *s*, *t*, *u* as derived from the match covers between the reference and each target (gray boxes). *k*-mers not contained by *I_r_* are not shared by all targets (dotted gray lines).

To determine which *k*-mers are shared between a set of target genomes, one target is chosen as the reference *r*, and all match covers, *M_rt_*, are intersected for each *t* in the target set. This intersection yields all matches shared by the target genomes relative to the sequence of the reference genome. Given the resulting match cover intersection *I_r_* for a collection of targets, a *k*-mer in *r* is shared by all other target genomes if, and only if, it is entirely contained within a single interval of *I_r_* (Figure 5).

**Figure 6:**
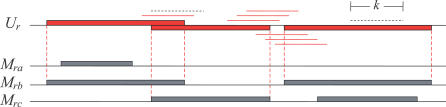
Unique *k*-mers (Red Lines) Obtained from a Union (*U_r_*) of Three Match Covers *M_ra_*, *M_rb_*, *M_rc_* *U_r_* intervals (red boxes) represent regions of the reference *r* matching some background genome *a*, *b*, *c* as derived from the match covers between the reference and each background (gray boxes). *k*-mers contained by *U_r_* match the background and are not unique (dotted gray lines).

A parallel stage of the signature pipeline computes a list of *k*-mers unique to a target genome with respect to some background. Once again, the match cover information is leveraged to efficiently identify these *k*-mers. Assuming the same target reference *r*, all match covers, *M_rb_*, are merged for each *b* in the background set. This produces a consolidated set of matches to the reference from the background. Matches smaller than *k*, and matches entirely contained by another interval, are irrelevant and can be removed. Given the resulting match cover union *U_r_* for a collection of backgrounds, a *k*-mer in *r* is unique with respect to the background if, and only if, it is not entirely contained within a single interval of *U_r_* (Figure 6). It is sufficient to compute unique *k*-mers with respect to a single target, because a sequence will only be reported as a signature if it is also shared by all target genomes. Thus, any single target is guaranteed to contain all of the ensuing signature sequences.

The interval set operations for signature detection are extremely efficient. For *M_RT_*-sorted reference-target match intervals and *T* target genomes, the time complexity for finding shared-mers is *O*(*M_RT_ log T*), with the log component incurred by a priority queue of overlapping interval end points. Given the bounded number of possible target genomes, this component can be treated as a constant and the complexity becomes linear. The time complexity for finding unique-mers is also linear: *O*(*M_RB_*) for *M_RB_* reference-background intervals. The results of these two operations are then intersected to identify sequence signatures, i.e., *k*-mers that are both shared by the targets and unique with respect to the background. Because all three of these operations are linear with regard to the number of match intervals and there cannot be more than *l* intervals for a sequence of length *l*, the complexity of extracting signatures from a match cover database is linear with regard to the size of the search space *O*(*l*). For a typical target and background set, this translates to about one minute of processing, given the current database size and computational processing speeds.

### Web interface.

The Insignia signature pipeline is accessible by a Web interface, hosted at the University of Maryland Center for Bioinformatics and Computational Biology (http://insignia.cbcb.umd.edu). This interface affords signature queries for any set of target genomes in the database, and displays results in the context of genome annotations for enhanced understanding and analysis. In addition, Insignia is closely coupled with the Gemina database (http://gemina.tigr.org), which provides sequence and annotation data for all bacterial, archaeal, and viral genomes available from GenBank, along with genotypic and epidemiological information for all NIAID category A,B,C pathogens.

To perform a signature query, the user specifies a reference genome, a set of target genomes, and a desired signature length. All reported signatures will be perfectly conserved among all genomes in the target set and absent from all other genomes. The reference genome, which is by definition one of the targets, serves as the coordinate system on which all signatures and genes (annotation) are based. Selection of the target genomes is carried out either through a list-based, tree-based, or query-based interface. In the list version, users are presented with a full listing of all genomes in the database, while the tree view arranges genomes in a taxonomy tree. The query interface available at the Gemina Web site facilitates text-based, controlled vocabulary queries of pathogen, host, disease, symptom, anatomy, transmission method, and geographic location attributes.

After computing all signatures for a given query, users may filter and display the results based on various experimental constraints. For instance, hybridization probes may require a certain GC content and melting temperature, so signatures falling below some user-specified thresholds can be screened out. Results may also be limited to specific genes, genes with specific functions, or intergenic sequence. After specifying the desired filters, signatures can be displayed and downloaded in tabular format or displayed in a genome browser, along with annotation data, to highlight each signature's position context.

To further support assay design, Insignia provides users with the ability to screen signatures for near matches and design signature-based primers. To search quickly for near matches, Insignia screens signature candidates against the National Center for Biotechnology Information (NCBI) databases using BLAST. This process helps eliminate signatures with near matches to background sequences and matches to sequences not included in the Insignia database, such as ESTs or environmental sequences. Once a set of signatures has been decided upon, the integrated Primer3 [22] software can be used to choose suitable primers and hybridization probes from the signatures.

### Assay design and validation.

The nucleotide sequences of the probes and primers for each TaqMan assay were selected from the signature set identified by Insignia for *V. cholerae O1 biovar El Tor strain N16961*. The probes and primers were designed outside of Insignia using commercially available design software (Allele ID, Premier Biosoft International, http://www.premierbiosoft.com). All assays were designed for PCR to run under the same conditions. The primers and probes were synthesized commercially (Invitrogen, http://www.invitrogen.com, and Sigma-Genosys/Sigma-Aldrich, http://www.sigmaaldrich.com). The probes were synthesized with the FAM fluorescent reporter dye at the 5′ end and with TAMRA quencher dye at the 3′ end. Genomic DNA was extracted from each inclusive and exclusive validation strain (DNeasy Blood and Tissue Kit, Qiagen, http://www.qiagen.com), and species identity was confirmed for each strain sample by partial 16S rDNA sequencing (MicroSeq ID, http://www.appliedbiosystems.com).

Real-time PCR was performed in a reaction mixture with a total volume of 25 μl containing 100 ng of genomic DNA, 500 nM of each primer, 250 nM of each fluorogenic probe, and TaqMan Universal Master Mix (Applied Biosystems). The Master Mix contained AmpErase uracil-N-glycosylase (UNG), deoxynucleoside triphosphate with dUTPs, ROX as an internal passive reference, and an optimized buffer component. Amplification and detection were carried out in an ABI 7500 Real-Time PCR System (Applied Biosystems) with an initial step of 50 °C for 2 min, 95 °C for 10 min, followed by 40 cycles of 95 °C for 15 s and 60 °C for 1 min.

All PCR assays were conducted in duplicate and Ct values were used to evaluate the extent to which each assay was inclusive of V. cholerae strains and/or excluded near-neighbor strains. Ct values of <21 were considered strong positive, and Ct values between 21 and 50 were binned in increments of 4 (i.e., 21–24, 25–28, etc.) to simplify analysis of the relative efficiency of PCR across all assays and strains.

## Supporting Information

Figure S1 Alternate Grayscale Version of Figure 3
(76 KB PDF)

Table S1 V. cholerae Assay Signature Sequences and Targeted Gene Function (100 KB PDF)

Table S2 Inclusive and Exclusive Strain Information (131 KB PDF)

Table S3 Detailed qPCR Results for All 3,450 Validation Experiments (89 KB PDF)
