# Supplementary material for: Comprehensive DNA Signature Discovery and Validation
Source: PLoS Comput Biol. 2007 May 18;3(5):e98. doi: 10.1371/journal.pcbi.0030098 (PMC1868776; doi:10.1371/journal.pcbi.0030098)
Supplement: Supplementary file 1 [file pcbi.0030098.sg001.pdf]

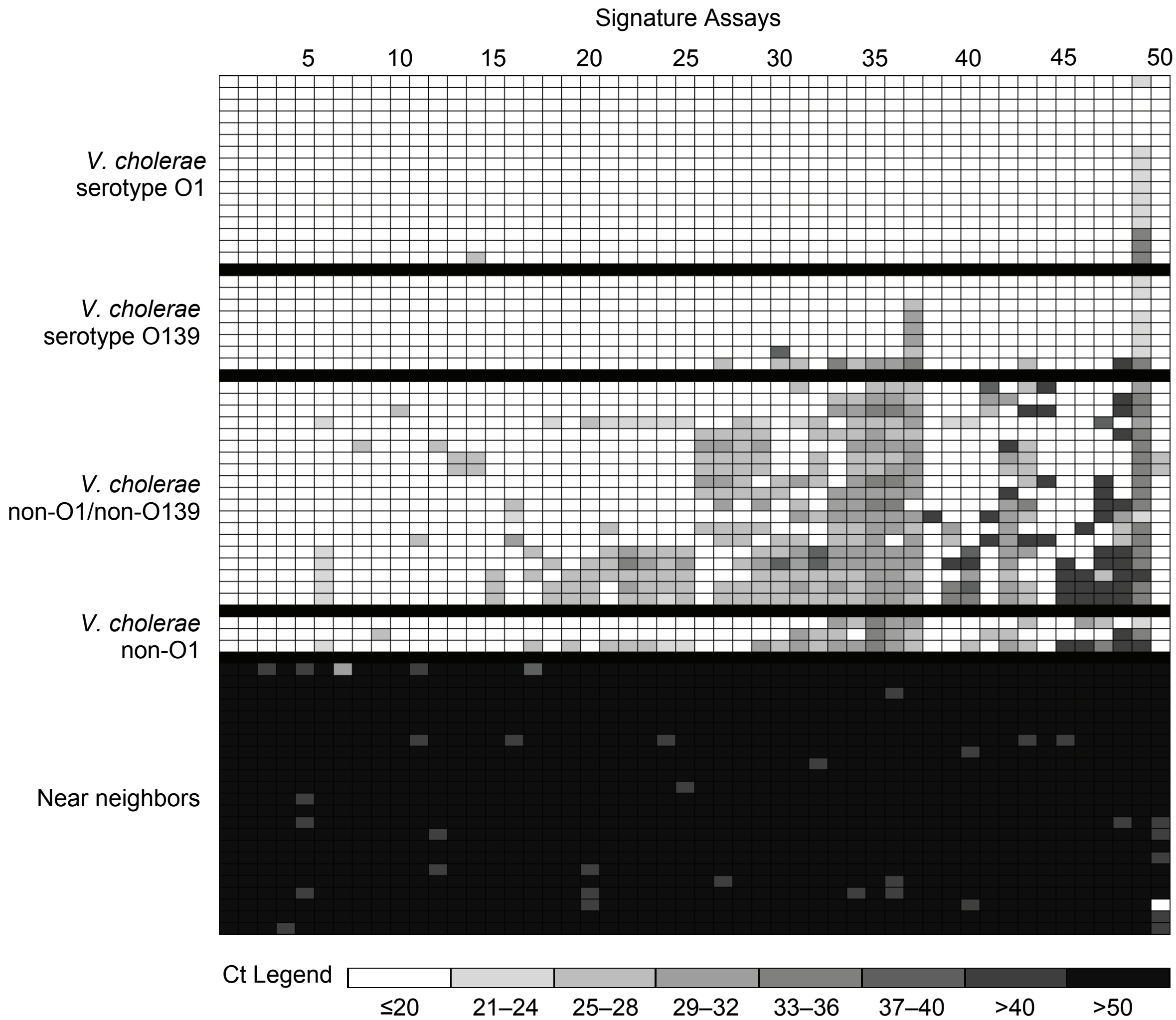

Signature Assays
5
10
15
20
25
30
35
40
45
50
V. cholerae serotype O1
V. cholerae serotype O139
V. cholerae non-O1/non-O139
V. cholerae non-O1
Near neighbors
Ct Legend
≤20
21–24
25–28
29–32
33–36
37–40
>40
>50
