# Supplementary material for: Comprehensive DNA Signature Discovery and Validation
Source: PLoS Comput Biol. 2007 May 18;3(5):e98. doi: 10.1371/journal.pcbi.0030098 (PMC1868776; doi:10.1371/journal.pcbi.0030098)
Supplement: Supplementary file 2 [file pcbi.0030098.st001.pdf]

**Reference strain from Insignia**			
Vibrio cholerae **Pacini N16961**		**biovar eltor, serovar O:1, serotype Inaba**	**stool from cholera patient, Bangladesh**
Inclusive strains			
**ID #**	**Scientific Name**	**Serogroup**	**Source**
NIH 42	*Vibrio cholerae*	O1	Mathbaria, Bangladesh clin. 2004
NIH 460	*Vibrio cholerae*	O1	Bakerganji, Bangladesh env. 2005
RC 121	*Vibrio cholerae*	O1	G.B. Nair; India, 1993, clinical
RC 144	*Vibrio cholerae*	O1	Mexico, first case, 1991
RC 145	*Vibrio cholerae*	O1	
RC 18	*Vibrio cholerae*	O1	Chile, 1991(?)
RC 25	*Vibrio cholerae*	O1	Mexico, 1991
RC 290	*Vibrio cholerae*	O1	Louisiana, 1978
RC 291	*Vibrio cholerae*	O1	From U.S. tourist returning from Cancun, Q. Roo, 1983
RC 610	*Vibrio cholerae*	O1	Tenanguillo, Edo. de Mexico
RC 618	*Vibrio cholerae*	O1	Tlaxcoapan, Hidalgo
RC 637	*Vibrio cholerae*	O1	Manzanillo, Colima
RC 72	*Vibrio cholerae*	O1	CDC, Cheryl Bopp/Joy Wells; received 3/12/05
RC 773	*Vibrio cholerae*	O1	Clinical isolate
RC 90	*Vibrio cholerae*	O1	Clinical, Lima, Peru; host for Peru phage 15
NIH 215	*Vibrio cholerae*	O139	Mathbaria, Bangladesh env. 2004
RC 106	*Vibrio cholerae*	O139	G.B. Nair; India, 1993, clinical
RC 30	*Vibrio cholerae*	O139	Bangladesh
RC 33	*Vibrio cholerae*	O139	India, 1985
RC 34	*Vibrio cholerae*	O139	Thailand, 8/25/1993
RC 36	*Vibrio cholerae*	O139	India, 1992 (P. Echererria); Stine et al., 2000
RC 37	*Vibrio cholerae*	O139	Zaire, 1978
RC 38	*Vibrio cholerae*	O139	Kenya
NIH 111	*Vibrio cholerae*	non-O1/non-O139	Mathbaria, Bangladesh env. 2004
NIH 172	*Vibrio cholerae*	non-O1/non-O139	Mathbaria, Bangladesh env. 2004
NIH 60	*Vibrio cholerae*	non-O1/non-O139	Mathbaria, Bangladesh env. 2004
NIH 63	*Vibrio cholerae*	non-O1/non-O139	Mathbaria, Bangladesh env. 2004
RC 242	*Vibrio cholerae*	non-O1/non-O139	I.G. Rivera, Brazil. Sewage
RC 380	*Vibrio cholerae*	non-O1/non-O139	Chesapeake Bay 98/102
RC 477	*Vibrio cholerae*	non-O1/non-O139	Chesapeake Bay 98/103
RC 525	*Vibrio cholerae*	non-O1/non-O139	Chesapeake Bay 98/101
RC 570	*Vibrio cholerae*	non-O1/non-O139	Chesapeake Bay 98/99
RC 572	*Vibrio cholerae*	non-O1/non-O139	Chesapeake Bay 98/100
RC 648	*Vibrio cholerae*	non-O1/non-O139	Chicoloapan, Edo. De Mexico
RC 651	*Vibrio cholerae*	non-O1/non-O139	Tampico, Tamaulipas
RC 678	*Vibrio cholerae*	non-O1/non-O139	Coatzacoalcos, Veracruz
RC 680	*Vibrio cholerae*	non-O1/non-O139	Veracruz,Veracruz
RC 69	*Vibrio cholerae*	non-O1/non-O139	I.G. Rivera; S. Paulo, marine sed
RC 708	*Vibrio cholerae*	non-O1/non-O139	Peru
RC 709	*Vibrio cholerae*	non-O1/non-O139	Peru
RC 710	*Vibrio cholerae*	non-O1/non-O139	Peru
RC 782	*Vibrio cholerae*	non-O1/non-O139	Fish from the Elbe River
RC 101	*Vibrio cholerae*	non-O1	Mexico, clinical
RC 60	*Vibrio cholerae*	non-O1	I.G. Rivera; Brazil, seawater
RC 63	*Vibrio cholerae*	non-O1	I.G. Rivera; S. Paulo, seawater
Exclusive strains			
**ID #**	**Scientific Name**	**Serogroup**	**Source**
RC 5	*Vibrio mimicus*		Ear, 35-year-old female, North Carolina
RC 255	*Vibrio aestuarianus*		Oyster, Oregon
RC 256	*Vibrio alginolyticus*	serotype XII	Spoiled horse mackerel which caused food poisoning, Japan
RC 257	*Vibrio fluvialis*	biotype 1	Human feces, Dacca, Bangladesh
RC 258	*Vibrio furnissii*		Human feces, Japan
RC 261	*Vibrio diazotrophicus*		Sea urchin gastrointestinal tract, Canada
RC 263	*Vibrio campbellii*		Seawater
RC 266	*Vibrio tubiashii*		Juvenile hard clams, Mercenaria mercenaria
RC 277	*Vibrio hollisae*		Human feces, Maryland
RC 304	*Vibrio parahaemolyticus*		Shirasu food poisoning, Japan
RC 745	*Vibrio metschnikovii*		Peru
RC 783	*Vibrio mediterranei*		CIP 107136
ATCC 10798	*Escherichia coli*		Feces from diphtheria convalescent
ATCC 14390	*Vibrio algosus*		
ATCC 25521	*Photobacterium leiognathi*		Light organ of teleostean fish in the family Leiognathidae
ATCC 25915	*Photobacterium angustum*		Seawater
ATCC 27390	*Vibrio abalonicus*		Wound infection of abalone
ATCC 29988	*Vibrio gazogenes*		Marine mud, Woods Hole, MA
ATCC 35912	*Vibrio cincinnatiensis*		Blood and cerebrospinal fluid, Ohio
ATCC 43979	*Aeromonas sobria*		Fish
ATCC 51183	*Vibrio navarrensis*		Sewage, Villafranca, Navarra, Spain
ATCC 51288	*Vibrio myliti*		Mussels, Mytilus edulis, Spain
ATCC 700797	*Vibrio aerogenes*		Seagrass bed sediment, Nanwan Bay, Taiwan, 1993
