# Supplementary material for: Comprehensive DNA Signature Discovery and Validation
Source: PLoS Comput Biol. 2007 May 18;3(5):e98. doi: 10.1371/journal.pcbi.0030098 (PMC1868776; doi:10.1371/journal.pcbi.0030098)
Supplement: Supplementary file 3 [file pcbi.0030098.st002.pdf]

Chromosome	Sequence	Gene	Probe Sequence	Forward Primer	Reverse Primer
1	89	Hypothetical Protein	TTCAGCGGCATCTTCAGGCACT	ATTATTTAGAAGCGATTGATGACG	GATATTGGTGCTAAACCTCTGG
1	727	Hypothetical Protein	ACCCACATATTCCGCACCGCCT	TTCTTGCAGTAAATCACTCAGG	TCTGTATTGGGAAATAGAGTAACG
1	1640	Hypothetical Protein	CAGCTCGATCAAGGCATTGCGC	CGGTAAATGAATGAGGATTGGG	CTTTCACCGATGAAGATGGC
1	1803	Inner Membrane Protein	AGCCCGACCACGATCACCGAT	AAGGTCATGATCTTCTGTTGC	GTGCGAGCATGTTCGTTATC
1	1951	Inner Membrane Protein	ACCGACTCCATCAGTGCCCAGT	CCAACCAAAGAATGGAGAGTG	CCCTATCCTTCTGCAAATGC
1	2572	Inner Membrane Protein	CGCAAAGCCCCTTCGCCGAGAGG	TCGATGGTGAATTTGAACCAATCC	CGCTTCTGGAGAGGTATAACGC
1	1483832	Deoxyribodipyrimidine photolyase, putative	CACGCCCGCTCGCCAATCCACA	ACTGGTCGATGCCTGTATGC	AGGAACAGTTGCGCCAAATAAG
1	1488652	Chemotaxis protein CheA	ACGCAAGTAGACCTCGTGCCGC	GGCTTTGTCATCGGGATTCG	AACTGTTACCTTGATGCTTGGG
1	1504110	VceA multidrug resistance protein	AGTGCAGTACCGCGCCGCCAA	AGGAACTACCGATCCACAAACC	AGTGCCCATCCTGACAGAGG
1	1556855	Vibrio cholerae Rtx toxin gene cluster	CTGGCGGCTGTCCTTTGCTTGCTC	TGGTTAGCGGTAGTGAGGATTTC	GCGGATGCACTGGACTTAGG
1	1569435	Hypothetical protein	CGCCACCCGATAGCAGCAGCCC	TCATGGTGTCGAGTTGCTGTG	CTAACCCAGACCAACCCAATCC
1	1589186	Hypothetical protein	TCAAGCCCGTTCCACCGTCGCA	TCCATGCACGGGTATATAAGGC	TGACAACGTATCGTAGAAACGC
1	1595270	Conserved hypothetical protein	ACTGTGTGGCCGCAAAACGCTTCC	AGGGAGTTTCGTAGAGATATCAGC	AGCCTCGTAAAGCGGAGTTG
1	1605417	Hypothetical protein	TGCACTGCTCCAGCTACCGGCCA	TCAAGGTTTTCGCCATCAAGATC	TTGCTGACATTGAATGGTGTAACC
1	1653990	DNA ligase	CGCCGAGCCGCCCAAGATTGGT	AACAAAACGGGCAAACCTTGG	CGGCAGTGGATTTAGTGATGTC
1	1667135	Conserved hypothetical protein	CCCCAAGCCGAGCGTGCGTGAA	CCGCCACAAAAGCCATTTCC	GATGCGTGGTTCCCAGAAGG
1	1677811	Conserved hypothetical protein	CGCCCCGATGACCACCAGCAGC	CACCGCAACGAATTCAACAGC	CCAAAGTAGGCCAGCAGTAAGC
1	1685459	Hypothetical protein	CCGCCCCAACCAGACAAAGCCC	TTGCTGCGAAAGGCGAAATAG	CTGCGTGAACCATTAAAACTCG
1	1693955	Conserved hypothetical protein	ATCGCCACCGAGCAAGCACGCT	ACATTCTGCGAGCGTTGGG	GCAGCAGGCTCCACAATCC
1	1700678	Acetolactate synthase	ATGCCAGCCACCAAGCCACTGC	TGCGTCGTATTACTCGGACTG	GGTGCCCACGACAGGTAAC
1	1703678	Conserved hypothetical protein	TTGACCAAGCTGCCGCCGCCT	CGAGGCCCTCAACAAAGTCC	AAGCTGTGAATCGATCCCGAAG
1	1714319	Hypothetical protein	ACCTAGCCCAGCCACCAGCCGT	CGGCAATATCCAGCCATCCC	CCGCCCAGCTTACAAAGCC
1	1716433	Response regulator	TTCACCTGCCTCGTCCGCCGTC	GGGCTGTTGTGCGGCTAG	ACGTTTACCGAAAGGCATTGAC
1	1719825	Conserved hypothetical protein	TTGGCTCCCACACTGACCGAGGGT	GCTGCAACAACAAAACAACTCG	AAAGTGTTACATCCTGATCAAGCC
1	1733322	Cadherin-related protein,authentic point mutation	TGCGACCGCTGTGGTGCCTTCA	CTGCTACTTGTTGCGTCACAG	CCAAAAGCAATGGCGATAATGC
1	1740118	Cadherin-related protein,authentic point mutation	TCGTCTGTGCCGCCGTTGCTGC	TGGGCTGCTGATGCCATTTAC	CACAGGTACAGCGACCATCAC
1	1750706	Na+/H+ antiporter protein	CCTACCGCCGCTATCGCAGGGA	TAATGCAGGAGCGAGCATACC	AACGTGAACTGCTGGAAGGAG
1	1764566	Sensor histidine kinase	ACGCTTCGCTCACGCTCGTTGT	ACTGGTCAGCCACTTTAGGC	CCGTGAACATGGGTTTCTTCG
1	1780451	Response regulator VieA	ACGCCGCTAAGCCCATGCCGAT	CAAGATTTGCCAGCCATCATCC	TTGGGGTAGATACTTGCCAAGG
1	1796491	ABC transporter, permease protein, putative	CCACCTTGATGCTCGGCGGCGG	GTCAGTCTGGCGCAGTATCTC	TTGGCAGAGGGCGTAAATGG
1	1814423	Peptide ABC transporter, permease protein	CCGCACTCGCCTTGGACGCTGA	ATGGTCGGCTACAGCTTAGC	TCCATCAGTTGCACCAAATAGC
1	1821759	Hypothetical protein	TGCCACAGTTGCCGGTGCTGCC	AGGTGAGCGTTAAGCAATCCG	GCCAAATTGCCCGTGAAACC
1	1830062	Conserved hypothetical protein	CGACCTGCCTCTGCCACCGCTG	CGCACTCGACGCAGTAGC	TCGCACACAATCCGTTTCTCC
1	1839488	Conserved hypothetical protein	CGACGCCACAGCCAACCAGAGC	AACACCACCAGAAACGCTTC	ACAAAGTATTGCCTGCGTTAGC
1	1878621	Hypothetical protein	TCCGCCACCAAACCCAGCCAGA	AGCGTTAGAAATGCTCGAATGG	GGGCAGGTAATGACTTCAACAC
1	1883005	Hypothetical protein	ACCTAACCACGCCACCATGCCG	GAATGCGAGGCTATTACCAAGG	GCAAATTGTGAAGCTGTGCTG
1	1888208	Paraquat-inducible protein A	TCCTTCTGTGGCACCTCGGCTCTT	GGCTCAAGCATTATGCAAGGG	CCAACCGAGTGCAAACATTTTG
1	1917029	Hypothetical protein	CTGCCTCTACCGCTTGCTCGCCC	AAGGTAAAATCGAATGCTGTTAGC	CGAATGTGATGACGCCAATTATG
2	113	Hypothetical Protein	TCGTTCCGCTTACGGCTCGCTT	GCCACGTTTTGAAGTGATGATG	TCAGAATGGCATTCACCGTAAC
2	1394	Hypothetical Protein	TGGTTTTGCCAGCCAGCCCCTC	GCGGTAGCTGTTGGATTGTTC	GTACCGAGATCAATTGGTTGGG
2	1436	Thiophene and furan oxidation protein	CAGCGGAAACCTTACCATCGGC	ACTGCGGCAAGATTATCTATG	ATTTCCCAGAAGAAGAGATCG
2	1525	Thiophene and furan oxidation protein	AGCATATCCATGACGACCGGGC	CTCCTTTAATCTGCAAAATACGG	GTGAAGATGTGCTTGAGCTG
2	1558	Hypothetical Protein	ACCGCCAGCCATGAAAGATGAGGC	CGCAAGTCAATCAACGTTTACTG	CTGCCCAGTTAGTCGGTGAG
2	2131	Hypothetical Protein	CCCTTCCCTTCGGAGAGACGACGA	ATACCGATGTAATGATGCTGAGTG	CCCAGAGATTGACCACAAACAG
2	2809	Hypothetical Protein	CAAAACCAACGCCCAGCCT	GAGCCTTAGATGAGATGG	CGGTTATTCGGTTCAATG
2	3502	Hypothetical Protein	CTTACGGTCGATTCCAGCTCTGCGC	TCAACTCCAAGTCACACTCAGG	GAGACGGATACCGAGAAGGC
2	3612	Hypothetical Protein	CGCACCGCAAGCACTCTGGC	CAATCGCTTTGCTTTGAGTGG	CGTCGCTGAATTACAACCTTTG
2	11500	malT Protein	ACCACCCCAGATTAGGCTTGTCGGC	GATCGTTATCGCTATCATCAATGC	AATTGGTGCTGTTTCGTTCCC
2	11638	Maltodextrin phosphorylase	CGGCAGATCCCACGGCATCGCT	CTGAGCTGAAGCAGGAAATTGG	AAACCGTATCACTTTGGTAGCC
2	12125	Hypothetical Protein	CACTACCAGACTCCTGTGCCTCTA	ATCCGAATCACTGCTATCTC	CTGCCTCTACGACTTTGG
