# Supplementary material for: Comprehensive DNA Signature Discovery and Validation
Source: PLoS Comput Biol. 2007 May 18;3(5):e98. doi: 10.1371/journal.pcbi.0030098 (PMC1868776; doi:10.1371/journal.pcbi.0030098)
Supplement: Supplementary file 4 [file pcbi.0030098.st003.pdf]

Gene	Hyp	Peptide ABC		Sensor	mioC	Hyp	IMP	IMP		acetolactate	Hyp				Na+/H+	ThdF		Conserved	Hyp		mioC	Hyp	Hyp	IMP	Hyp
	Prot	sporter, permease	Con Hyp Prot	histidine kinase	Prot	Prot	Prot	Prot	Con hyp Prot	synthase	Prot	DNA ligase	Con hyp Prot	Hyp Prot	antiporter prot		Hyp Prot	Hyp Prot	Prot	Con Hyp Prot	Prot	Prot	Prot	Prot	Prot
CHROMOSOME	2	1	1	1	1	1	1	1	1	1	1	1	1	1	1	2	1	1	2	1	1	2	2	1	2
ID #	3502	1814423	1830062	1764566	727	2131	1951	1803	1667135	1700678	1640	1653990	1677811	1605417	1750706	1436	1821759	1595270	113	1719825	89	1394	1558	2572	3612
Vibrio cholerae N16961	15.16	15.17	15.43	15.64	19.1	18.1	16.76	17.1	15.37	16.41	16.07	15.51	15.53	16.31	15.32	16.9	15.58	15.93	16.3	15.66	14.99	16.5	15.41	18.96	15.17
NIH 42	15.01	15.37	15.55	15.75	16.8	15.02	15.11	15.44	15.31	16.17	14.62	15.95	15.56	16.34	15.46	16.26	15.68	15.69	14.85	15.85	14.91	15.09	15.11	15.57	14.67
NIH 460	15.03	15.16	15.34	15.5	16.92	15.22	15.17	15.4	15.32	16.17	14.65	15.84	15.45	16.29	15.22	16.46	15.39	15.66	14.89	15.58	15.03	15.07	15.12	15.56	14.62
RC 144	15.23	15.39	15.52	15.68	16.93	15.47	15.16	15.47	15.44	16.3	14.81	16.11	15.78	16.61	15.34	16.05	15.53	15.98	15.08	15.91	15.09	15.29	15.32	15.85	14.86
RC 610	15.14	15.41	15.58	15.76	17.14	15.43	15.28	15.57	15.66	16.52	14.82	16.24	15.86	16.7	15.47	16.5	15.72	16.03	15.03	15.94	15.09	15.24	15.3	15.77	14.85
RC 637	15.13	15.24	15.42	15.6	16.95	15.41	15.15	15.55	15.38	16.24	14.77	15.95	15.6	16.5	15.3	16.11	15.39	15.84	14.99	15.76	15.08	15.23	15.27	15.76	14.77
RC 121	15.3	15.19	15.49	15.68	17.02	15.45	15.25	15.56	15.53	16.38	14.84	16.15	15.82	16.63	15.34	16.18	15.5	16	15.06	15.88	15.18	15.34	15.37	15.83	14.84
RC 145	15.28	15.38	15.56	15.71	17.14	15.59	15.26	15.51	15.42	16.34	14.83	16.03	15.65	16.61	15.37	16.26	15.53	15.93	15.11	15.98	15.12	15.3	15.37	15.86	14.87
RC 18	15.21	15.66	15.84	16.08	17.33	15.48	15.5	15.61	15.81	16.6	14.81	16.41	16.1	16.72	15.76	16.65	16.04	16.18	15.17	16.23	15.18	15.34	15.34	15.92	14.9
RC 25	15.13	15.26	15.39	15.52	17.25	15.43	15.31	15.5	15.56	16.35	14.79	16.14	15.76	16.64	15.27	16.39	15.44	16.07	15.25	15.77	15.09	15.24	15.31	15.76	14.8
RC 618	15.19	15.33	15.46	15.65	17.11	15.4	15.28	15.52	15.46	16.28	14.75	16	15.62	16.51	15.32	16.34	15.54	15.89	15.05	15.8	15.08	15.23	15.29	15.72	14.77
RC 773	15.25	15.4	15.58	15.84	16.95	15.58	15.34	15.73	15.66	16.55	14.94	16.36	16.07	16.8	15.51	16.19	15.69	16.19	15.15	16.01	15.23	15.41	15.5	16.01	14.97
RC 90	15.22	15.32	15.47	15.68	17.08	15.41	15.26	15.56	15.44	16.26	14.79	16.06	15.66	16.56	15.35	16.28	15.54	15.88	15.08	15.82	15.12	15.29	15.34	15.883	14.9
RC 290	15.29	15.35	15.54	15.75	17.16	15.62	15.31	15.56	15.65	16.53	14.81	16.31	15.97	16.96	15.41	16.3	15.58	16.21	15.2	16	15.14	15.39	15.41	15.97	14.91
RC 72	15.11	15.31	15.45	15.68	17.15	15.36	15.32	15.58	15.53	16.42	14.77	16.18	15.85	16.71	15.38	16.53	15.59	16	15.01	15.8	15.09	15.24	15.24	15.77	14.72
RC 291	15.36	16.54	15.72	15.98	17.32	15.81	15.39	15.63	15.86	16.7	14.87	16.49	16.29	26.43	15.64	16.71	16.9	16.34	15.17	16.25	15.19	15.39	15.46	16.2	14.92
RC 38	15.27	15.32	15.53	15.58	17.2	15.77	15.53	15.72	15.55	17.03	14.86	16.27	15.89	16.46	15.36	16.48	15.48	16.03	15.19	15.86	15.22	15.33	15.37	16.09	14.9
RC 37	15.19	15.24	15.34	15.52	17.19	15.49	15.29	15.54	15.38	16.2	14.76	16.07	15.66	16.59	15.23	16.26	15.36	15.86	15.08	15.74	15.09	15.3	15.26	15.84	14.82
NIH 215	14.96	15.27	15.43	15.66	16.96	15.17	15.21	15.43	15.24	16.03	14.64	15.72	15.31	16.18	15.38	16.44	15.58	15.55	14.81	15.75	14.94	15.06	15.1	15.6	14.72
RC 33	15.51	15.68	15.86	16.08	17.59	15.91	15.63	15.92	15.76	16.64	15.13	16.38	16.08	16.99	15.66	16.65	15.85	16.35	15.37	16.25	15.49	15.69	15.75	16.35	15.16
RC 34	15.26	15.36	15.5	15.64	17.04	15.49	15.35	15.54	15.37	16.47	14.83	15.97	15.96	16.58	15.31	16.17	15.58	15.85	15.12	15.82	15.13	15.33	15.34	17.19	14.86
RC 36	15.22	15.25	15.4	15.53	17.04	15.44	15.26	15.52	15.57	16.22	14.77	16.28	15.58	16.52	15.26	16.14	15.45	16.13	15.08	15.75	15.1	15.45	15.27	15.81	14.91
RC 30	15.18	15.26	15.44	15.63	17.06	15.38	15.25	15.53	15.95	16.81	14.74	16.64	16.31	17.09	15.33	16.16	15.52	16.41	15.06	15.77	15.11	15.27	15.31	15.77	14.83
RC 106	15.41	16.83	16.04	17.59	18.05	15.65	15.49	15.88	16.18	16.59	14.88	16.99	16.02	16.91	15.59	20.42	16.46	16.23	15.33	16.17	15.32	15.52	15.53	16.1	15.01
RC 648	15.24	15.27	15.41	15.68	16.93	15.43	15.3	16.07	18.03	16.5	14.79	16.97	15.76	16.53	16.71	16.19	16.51	15.96	15.05	16.03	15.1	15.26	15.29	15.86	14.86
NIH 60	20.26	15.41	17.01	16.77	16.85	15.31	15.12	15.42	17.87	16.14	14.52	15.92	15.51	16.7	15.73	16.18	16.84	15.77	14.86	16.32	14.97	15.13	15.17	15.66	14.73
RC 69	20.15	14.73	14.84	15.02	17.16	15.36	15.46	15.8	15.74	20.43	15.03	16.33	16.01	16.71	14.69	16.44	14.91	16.17	14.97	16.22	15.28	15.16	15.24	16.83	14.75
NIH 63	15.37	15.5	15.49	15.75	17.02	22.84	15.34	16.67	15.74	16.06	14.6	15.75	15.4	18.9	15.41	16.3	16.48	20.86	17.89	21.25	22.33	22.62	23	24.71	21.64
NIH 111	15.08	15.38	15.8	15.8	17.08	15.33	15.23	15.5	15.36	16.1	14.68	16.56	15.4	16.28	15.49	16.43	16.73	15.69	14.97	16.02	15.06	16.05	15.18	16.82	14.75
RC 570	15.28	15.33	15.51	15.83	17.29	15.48	15.63	28.34	15.76	16.45	14.82	22.84	15.79	16.75	15.61	16.85	16.65	16.04	15.18	16.62	15.24	15.42	15.52	16	15.04
RC 525	15.11	15.27	15.5	15.68	16.94	15.27	15.22	15.41	15.46	16.23	14.58	15.91	26.1	24.31	15.37	16.35	16.63	15.78	15	16.39	15.81	15.2	15.24	16.9	14.66
RC 380	15	15.44	15.65	15.86	16.68	15.27	15.17	15.32	15.35	16.15	14.56	15.91	27.08	24.26	15.59	16.1	16.75	15.72	14.89	16.54	15.69	15.13	15.15	16.75	14.67
RC 572	15.2	15.33	15.48	15.7	17.09	15.38	15.38	15.59	15.64	16.38	14.66	16.15	15.89	17.02	15.77	16.49	17.11	16.02	15.04	16.17	15.16	15.3	15.33	15.95	14.88
RC 477	15.02	15.4	15.65	15.76	16.74	15.2	15.09	15.41	17.51	16.78	14.64	16.82	15.58	16.72	15.68	17.79	16.5	15.71	14.88	15.96	15.67	15.08	15.08	15.59	14.65
RC 242	15.28	16.39	15.32	17.18	16.96	15.43	15.26	15.6	15.44	16.18	14.68	16.02	15.57	16.18	15.26	20.47	15.43	15.91	15.17	15.75	15.1	15.28	15.32	15.79	14.86
RC 678	15.59	15.48	17.23	15.84	17.34	15.62	15.51	16.45	16.54	16.87	14.88	17.55	16.19	16.97	15.77	22.83	17.05	16.32	15.23	16.01	16.28	15.51	15.48	16.03	15.05
RC 782	15.4	15.4	15.55	15.78	17.05	15.51	15.38	15.78	15.61	16.39	14.73	16.19	15.79	18.1	15.8	16.87	16.79	16.02	15.07	16.47	26.19	15.27	15.38	15.86	14.73
NIH 172	20.46	15.44	15.55	15.9	17.17	15.26	15.29	16.24	18.3	16.24	25.78	16.09	15.67	16.45	15.8	29.01	16.98	15.81	14.91	16.31	15.05	15.11	15.15	15.66	14.71
RC 680	15.57	16.49	16.61	15.68	17.04	23.47	16.56	15.8	15.89	16.68	14.87	16.56	16.15	19.44	15.38	16.32	28.6	16.31	18.27	16.07	27.24	29.45	28.37	25.59	26.59
RC 710	15.4	15.4	15.58	15.69	16.87	23.24	15.39	17.54	16	16.38	14.72	16.12	16.09	19.03	15.35	16.14	16.34	28.99	17.61	29.44	27.43	34.66	30.04	26.23	28.62
RC 651	15.59	16.39	15.44	15.64	17.05	23.12	16.43	15.79	15.51	16.37	14.84	16.15	16.01	19.03	20.24	20.02	27.61	16.02	26.78	28.03	25.88	27.57	27.18	25.07	25.73
RC 708	15.33	15.38	16.15	15.66	18.04	23.04	15.34	17.58	15.58	16.43	14.65	16.2	15.93	16.34	20.68	16.11	16.2	28.38	26.73	28.04	15.07	28.96	27.94	25.29	26.78
RC 709	15.23	15.22	15.92	15.42	17.86	23.2	15.17	17.35	15.33	16.16	14.52	15.87	15.55	16.12	20.31	16.02	15.83	28.75	26.36	28.72	14.93	28.65	27.58	24.93	26.24
RC 63	15.42	15.58	16.09	16.08	17.15	15.65	15.49	15.85	16.47	16.9	14.87	16.65	16.54	17.13	15.71	16.29	16.87	16.41	15.23	16.28	15.25	15.26	15.6	16.08	15.06
RC 60	15.43	15.38	15.56	15.75	16.99	15.44	15.25	15.6	27.44	16.32	14.86	16.63	15.73	16.67	15.41	16.18	15.58	16.01	15.12	16.09	15.18	15.3	15.36	15.89	14.8
RC 101	15.41	16.32	15.42	15.59	16.86	22.91	16.18	15.54	15.42	16.3	14.74	16.05	15.92	16.25	19.97	19.49	26.36	15.93	25.62	16.17	25	25.86	25.71	24.49	24.28

EXCLUSIVE ASSAYS	3502	1814423	1830062	1764566	727	2131	1951	1803	1667135	1700678	1640	1653990	1677811	1605417	1750706	1436	1821759	1595270	113	1719825	89	1394	1558	2572	3612
Vibrio mimicus	>50	>50	41.02	>50	48.65	>50	33.04	>50	>50	>50	48.83	>50	>50	>50	>50	>50	37.06	>50	>50	>50	>50	>50	>50	>50	>50
Vibrio aestuarianus	>50	>50	>50	>50	>50	>50	>50	>50	>50	>50	>50	>50	>50	>50	>50	>50	>50	>50	>50	>50	>50	>50	>50	>50	>50
Vibrio alginolyticus	>50	>50	>50	>50	>50	>50	>50	>50	>50	>50	>50	>50	>50	>50	>50	>50	>50	>50	>50	>50	>50	>50	>50	>50	>50
Vibrio fluvialis	>50	>50	>50	>50	>50	>50	>50	>50	>50	>50	>50	>50	>50	>50	>50	>50	>50	>50	>50	>50	>50	>50	>50	>50	>50
Vibrio furnissii	>50	>50	>50	>50	>50	>50	>50	>50	>50	>50	>50	>50	>50	>50	>50	>50	>50	>50	>50	>50	>50	>50	>50	>50	>50
Vibrio diazotrophicus	>50	>50	>50	>50	>50	>50	>50	>50	>50	>50	>50	>50	>50	>50	>50	>50	>50	>50	>50	>50	>50	>50	>50	>50	>50
Vibrio campbellii	>50	>50	>50	>50	>50	>50	>50	>50	>50	>50	49.17	>50	>50	>50	>50	44.31	>50	>50	>50	>50	>50	>50	>50	42.07	>50
Vibrio tubiashii	>50	>50	>50	>50	>50	>50	>50	>50	>50	>50	>50	>50	>50	>50	>50	>50	>50	>50	>50	>50	>50	>50	>50	>50	>50
Vibrio hollisae	>50	>50	>50	>50	>50	>50	>50	>50	>50	>50	>50	>50	>50	>50	>50	>50	>50	>50	>50	>50	>50	>50	>50	>50	>50
Vibrio parahaemolyticus	>50	>50	>50	>50	>50	>50	>50	>50	>50	>50	>50	>50	>50	>50	>50	>50	>50	>50	>50	>50	>50	>50	>50	>50	>50
Vibrio metschnikovii	>50	>50	>50	>50	>50	>50	>50	>50	>50	>50	>50	>50	>50	>50	>50	>50	>50	>50	>50	>50	>50	>50	>50	>50	48.8
Vibrio mediterranei	>50	>50	>50	>50	43.1	>50	>50	>50	>50	>50	>50	>50	>50	>50	>50	>50	>50	>50	>50	>50	>50	>50	>50	>50	>50
E. coli 10798	>50	>50	>50	>50	>50	>50	>50	>50	>50	>50	>50	>50	>50	>50	>50	>50	>50	>50	>50	>50	>50	>50	>50	>50	>50
Vibrio algosus	>50	>50	>50	>50	44.7	>50	>50	>50	>50	>50	>50	>50	>50	>50	>50	>50	>50	>50	>50	>50	>50	>50	>50	>50	>50
Photobacterium leiognathi	>50	>50	>50	>50	>50	>50	>50	>50	>50	>50	>50	47.64	>50	>50	>50	>50	>50	>50	>50	>50	>50	>50	>50	>50	>50
Photobacterium angustum	>50	>50	>50	>50	>50	>50	>50	>50	>50	>50	>50	>50	>50	>50	>50	>50	>50	>50	>50	>50	>50	>50	>50	>50	>50
Vibrio abalonicus	>50	>50	>50	>50	>50	>50	>50	>50	>50	>50	>50	>50	>50	>50	>50	>50	>50	>50	>50	>50	>50	>50	>50	>50	>50
Vibrio gazogenes	>50	>50	>50	>50	>50	>50	>50	>50	>50	>50	>50	47.82	>50	>50	>50	>50	>50	>50	>50	48.9	>50	>50	>50	>50	>50
Vibrio cincinnatiensis	>50	>50	>50	>50	>50	>50	>50	>50	>50	>50	>50	>50	>50	>50	>50	>50	>50	>50	>50	>50	>50	>50	>50	>50	>50
Aeromonas sobria	>50	>50	>50	>50	44.08	>50	>50	>50	>50	>50	>50	>50	>50	>50	>50	>50	>50	>50	>50	44.09	>50	>50	>50	>50	>50
Vibrio navarrensis	>50	>50	>50	>50	>50	>50	>50	>50	>50	>50	>50	>50	>50	>50	>50	>50	>50	>50	>50	43.38	>50	>50	>50	>50	>50
Vibrio mytili	>50	>50	>50	>50	>50	>50	>50	>50	>50	>50	>50	>50	>50	>50	>50	>50	>50	>50	>50	>50	>50	>50	>50	>50	>50
Vibrio aerogenes	>50	>50	>50	41.21	>50	>50	>50	>50	>50	>50	>50	>50	>50	>50	>50	>50	>50	>50	>50	>50	>50	>50	>50	>50	>50

LEGEND	O1	O139	non-O1/non-O139	non-O1		<20	21-24	25-28	29-32	33-36	37-39	>40	>50

Gene	VceA	Chemotaxis protein CheA	Hyp Prot	Rtx toxin	Con Hyp prot	Maltodextrin phosphorylase	Con Hyp Prot	Hyp Prot	Cadherin related prot	Hyp Prot	Cadherin related prot	Hyp Prot	ABC transporter	ThdF	Hyp Prot	Response Regulator	Hyp Prot	Response regulator VieA	Hyp prot	Con Hyp Prot	malT	Hyp Prot	Paraquat inducible prot A	Hyp Prot	oxyribodipyrimidi photolyase
CHROMOSOME	1	1	1	1	1	2	1	1	1	1	1	1	1	2	2	1	1	1	1	1	2	1	1	2	1
ID #	1504110	1488652	1714319	1556855	1703678	11638	1693955	1883005	1740118	1569435	1733322	1917029	1796491	1525	2809	1716433	1878621	1780451	1685459	1839488	11500	1589186	1888208	12125	1483832
Vibrio cholerae N16961	15.8	15.4	15.81	15.34	15.44	15.54	16	15.49	15.43	16.1	15.94	15.78	15.25	16.83	18.22	15.24	15.63	15.76	15.49	15.09	15.31	15.19	15.64	22.35	17.26
NIH 42	15.81	15.26	15.76	15.19	15.02	15.36	15.73	15.32	15.25	16.14	16.23	15.38	15.07	15.02	16.66	15.38	15.76	15.92	15.36	15.14	15.03	14.91	15.37	20.07	15.74
NIH 460	15.8	15.21	15.54	15.14	15.08	15.35	15.74	16.7	14.97	16.15	15.98	16.74	14.88	15.05	17.03	15.18	17.09	15.66	15.34	14.95	15.09	14.97	16.82	20.01	15.67
RC 144	16.1	15.44	15.6	14.91	15.51	15.56	15.98	15.54	15.52	16.43	16.13	15.58	15.1	15.13	16.08	15.35	15.93	15.89	15.45	15.03	15.34	15.1	15.6	20.9	16.06
RC 610	16.17	15.54	15.79	15.42	15.4	15.55	16.15	15.61	15.28	16.34	16.27	15.67	15.08	15.19	16.77	15.38	15.93	15.99	15.66	15.19	15.34	15.14	15.61	20.93	16.01
RC 637	16.1	15.46	15.59	15.38	15.2	15.48	15.87	15.42	15.29	15.87	16.13	15.52	15.01	15.14	16.2	15.25	16.03	15.75	15.38	14.86	15.21	15.06	15.57	20.54	15.92
RC 121	16.17	15.46	15.66	15.41	15.76	15.68	16.1	15.52	15.2	16.46	16.12	15.6	15.07	15.18	16.12	15.3	16.02	15.87	15.49	15.08	15.37	15.14	15.55	21.41	16.02
RC 145	16.16	15.51	15.69	15.43	15.35	15.57	16	16.99	15.29	16.66	16.19	17.23	15.14	15.16	16.73	15.39	17.03	15.94	15.44	15.59	15.27	15.13	16.81	21.36	16.01
RC 18	16.28	15.64	16.1	15.53	16.22	15.69	16.32	16.07	15.48	16.71	16.57	16.18	15.31	15.19	16.72	15.79	16.44	16.28	15.72	15.2	15.39	15.3	15.97	21.11	16.23
RC 25	16.2	15.48	15.62	15.32	18.07	15.61	16.08	15.49	15.15	16.51	16.04	15.59	14.98	15.16	16.43	15.27	15.97	15.81	15.53	15.05	15.31	15.08	15.55	21.27	15.99
RC 618	16.03	15.41	15.62	15.3	15.21	15.53	15.93	15.42	15.2	15.87	16.11	15.5	15.04	15.14	16.36	15.33	15.87	15.85	15.41	15.16	15.27	15.05	15.47	22	15.89
RC 773	16.16	15.63	15.75	15.55	15.51	15.72	16.21	15.67	15.34	15.98	16.27	15.73	15.13	15.27	16.11	15.46	16.16	15.98	15.66	15.06	15.42	15.21	15.77	21.16	16.11
RC 90	16.08	15.38	15.68	15.34	15.17	15.56	15.96	15.5	15.2	16.43	16.12	15.57	15.05	15.17	16.29	15.33	15.92	15.9	15.45	15.04	15.31	15.1	15.52	21.19	15.97
RC 290	16.22	15.53	15.72	15.53	15.63	15.69	16.2	15.6	15.39	16.15	16.27	15.6	15.13	15.18	16.26	15.42	15.97	15.96	15.61	15.44	15.36	15.29	15.72	34.15	16.09
RC 72	16.07	15.4	15.67	15.32	15.18	15.57	16.05	15.62	15.22	15.98	16.19	15.7	15	15.15	17.02	15.34	15.84	15.86	15.55	15.8	15.22	15.12	15.44	33.2	15.95
RC 291	16.04	15.37	16.02	15.73	15.74	15.85	16.38	15.97	15.54	16.28	16.46	16.14	15.48	15.24	16.38	15.71	16.36	16.27	16.17	15.59	15.42	15.41	15.91	34.95	15.91
RC 38	16.39	15.74	15.61	15.74	15.43	15.89	16.05	15.67	15.21	16.59	16.13	15.61	15.03	15.26	16.46	15.39	15.9	15.89	15.52	15.44	15.39	15.23	15.69	22.53	16.3
RC 37	16.2	15.53	15.59	15.44	15.4	15.59	15.9	15.58	15.1	16.58	16.06	15.55	14.97	15.17	16.28	15.28	16.04	15.78	15.4	15.05	15.31	15.1	15.67	21.7	16
NIH 215	15.77	15.21	15.69	15.15	14.94	15.36	15.63	15.32	15.18	16.2	16.21	27.37	14.99	14.94	17.09	15.34	15.77	15.8	15.29	15.06	15.06	14.91	15.39	20.21	15.7
RC 33	16.68	16.02	16.05	16.04	18.73	16.07	16.31	17.03	15.51	16.36	16.55	31.07	15.35	15.52	16.48	15.71	17.18	16.28	15.74	15.4	15.76	15.4	16.88	22.39	16.55
RC 34	16.3	15.51	15.64	15.47	15.48	15.67	16.16	15.49	15.22	15.75	16.14	30.4	15.05	15.13	16.46	15.39	15.94	15.86	15.6	15.15	15.38	15.07	15.55	21.06	16.08
RC 36	16.08	15.45	15.51	15.38	15.28	15.6	15.92	15.49	15.16	16.49	16.04	29.6	15.01	15.17	16.12	15.27	15.95	15.82	15.39	15.01	15.3	15.08	15.55	21.12	15.95
RC 30	16.04	15.41	15.62	15.33	37.52	15.55	16.5	15.54	15.19	15.98	16.11	28.35	15.01	15.16	16.28	15.32	15.81	15.82	15.95	15.04	15.31	15.1	15.51	21.36	15.93
RC 106	16.34	28.85	16.41	15.68	29.03	28.91	16.22	33.46	28.85	29.93	30.23	34.05	16.36	15.84	16.33	15.57	18.54	29.84	15.68	15.34	15.5	15.33	>40	35.04	16.14
RC 648	16.17	15.48	17.63	15.44	15.49	26.74	16.03	15.51	19.55	27.85	28.31	27.07	15.18	15.18	16.26	35.73	15.79	22.04	>40	15.3	15.29	15.15	15.79	32.72	16.06
NIH 60	16.01	15.38	18.23	17.84	15.67	15.43	15.77	28.4	28.82	30.31	30.02	28.79	15.24	15.04	16.37	34.06	29.25	16.03	16.42	15.55	15.47	14.99	>40	33.46	16.16
RC 69	16.25	15.75	15.75	15.54	15.84	15.46	16.35	30.22	30.66	33.13	33.21	30.18	14.48	15.25	16.5	21.3	15.98	>40	>40	14.49	15.19	15.27	>40	33.44	16.23
NIH 63	15.91	15.35	20.67	24.64	19.87	24.55	20.81	20.32	20.41	25.95	21.64	29.52	17.27	22.62	24.06	16.13	20.87	15.91	15.31	20.84	15.13	14.98	19.18	32.04	18.88
NIH 111	28.41	27.81	27.94	27.83	15.42	15.45	20.75	27.38	28.13	29.36	29.4	28.86	15.25	15.1	16.67	15.55	15.76	16.31	16.55	15.4	15.08	15.01	>40	33.63	15.9
RC 570	29.48	29.55	27.58	29.96	15.64	15.73	16.09	15.74	31.07	32.98	32.74	29.79	17.32	15.33	17.48	15.47	>40	25.24	16.07	15.26	15.59	15.26	16.04	33.72	16.04
RC 525	27.35	26.89	27.42	26.68	15.14	15.47	20.15	16.02	27.53	28.96	28.27	31.87	15.11	14.96	16.8	15.36	31.56	22.91	15.83	16.2	15.14	15.13	16.24	34.14	27.37
RC 380	28.13	27.45	28.1	27.71	15.22	15.46	20.08	15.4	28.4	29.26	29.55	29.2	15.25	14.91	16.33	15.52	28.61	22.65	15.69	15.25	15.08	15	15.72	33.18	28.16
RC 572	30.65	31.67	28.46	15.4	28.87	29.57	20.28	15.62	29.04	36.17	33.82	29.89	17.11	15.16	16.9	15.41	16	23.57	>40	16.2	15.55	>40	15.91	33.41	19.1
RC 477	27.78	27.09	28.43	28.89	26.36	15.38	19.71	29.22	30.16	31.16	31.05	30.33	15.24	15.02	16.29	15.47	>40	16.01	15.63	15.69	14.99	>40	15.55	33.13	18.54
RC 242	16.09	29.85	16.01	30.41	17.88	28.8	15.87	29.54	32.6	35.14	34.97	15.57	15.95	15.48	16.31	15.88	32.27	33.18	15.72	15.24	15.31	>40	>40	33.93	16.02
RC 678	16.37	15.7	16.46	15.66	17.78	30.38	20.81	30.11	31.12	32.23	32.55	30.67	>40	15.36	17.24	>40	30.89	24.5	16.37	16.09	15.52	>40	32.16	21.75	16.24
RC 782	28.02	27.54	27.96	27.72	15.51	15.66	16.05	28.18	28.91	29.49	30.18	28.61	15.51	30.1	16.77	15.4	29.17	16.31	16.01	15.5	>40	15.19	28.72	34.01	15.88
NIH 172	16.07	28.5	28.86	15.43	27.86	27.57	15.93	28.78	29.23	30.64	16.57	30.42	15.24	26.44	17.57	>40	29.82	>40	>40	15.54	15.2	15.19	28.5	33.65	16.37
RC 680	16.36	16.01	16.2	28.8	29.3	29.69	30.37	29.58	30.08	31.16	31.42	29.66	16.12	15.7	38.86	16.13	30.34	30.62	15.78	15.19	15.47	>40	>40	33.38	16.53
RC 710	16.11	15.8	28.82	30.58	30.42	30.46	31.26	30.76	30.97	32.06	32.27	30.91	17.1	>40	>40	16.03	31.49	15.81	15.55	>40	15.3	>40	>40	33.66	18.39
RC 651	16.27	15.87	16.13	28.18	22.65	28.8	28.06	28.02	28.94	30.35	30.41	28.04	15.98	15.64	32.69	15.95	28.81	15.88	15.53	>40	>40	27.3	>40	>40	16.47
RC 708	16.09	27.9	28.12	28.68	28.96	29.1	29.65	29.16	29.28	30.34	30.77	29.31	16.03	33.58	37.35	16	29.98	22.23	16.54	>40	>40	>40	>40	33.38	15.89
RC 709	15.87	28.23	28.32	28.14	28.15	28.25	28.92	28.15	28.43	29.5	29.81	28.36	15.77	36.7	35.73	15.66	29.14	21.48	16.3	>40	>40	>40	>40	33.39	15.74
RC 63	16.49	15.84	16.41	15.71	17.4	15.9	16.55	30.01	28.86	34.25	31.2	30.21	15.31	15.35	16.23	15.73	16.22	21.83	16.26	15.5	29.92	28.21	16.08	21.36	16.63
RC 60	16.2	15.55	17.5	15.5	15.47	30.53	20.21	28.33	15.33	33.5	29.5	28.35	15.2	15.18	16.25	29.77	28.91	15.94	15.83	15.26	15.29	15.16	>40	34.25	16.1
RC 101	16.14	15.66	16.05	30.07	28.62	29.81	26.71	31.08	31.2	34.85	32.85	26.21	16.91	15.47	28.37	15.83	30.42	21.32	15.42	>40	>40	30.91	>40	>40	16

EXCLUSIVE ASSAYS	1504110	1488652	1714319	1556855	1703678	11638	1693955	1883005	1740118	1569435	1733322	1917029	1796491	1525	2809	1716433	1878621	1780451	1685459	1839488	11500	1589186	1888208	12125	1483832
Vibrio mimicus	>50	>50	>50	>50	>50	>50	>50	>50	>50	>50	>50	>50	>50	>50	>50	>50	>50	>50	>50	>50	>50	>50	>50	>50	>50
Vibrio aestuarianus	>50	>50	>50	>50	>50	>50	>50	>50	>50	>50	>50	>50	>50	>50	>50	>50	>50	>50	>50	>50	>50	>50	>50	>50	>50
Vibrio alginolyticus	>50	>50	>50	>50	>50	>50	>50	>50	>50	>50	43.44	>50	>50	>50	>50	>50	>50	>50	>50	>50	>50	>50	>50	>50	>50
Vibrio fluvialis	>50	>50	>50	>50	>50	>50	>50	>50	>50	>50	>50	>50	>50	>50	>50	>50	>50	>50	>50	>50	>50	>50	>50	>50	>50
Vibrio furnissii	>50	>50	>50	>50	>50	>50	>50	>50	>50	>50	>50	>50	>50	>50	>50	>50	>50	>50	>50	>50	>50	>50	>50	>50	>50
Vibrio diazotrophicus	>50	>50	>50	>50	>50	>50	>50	>50	>50	>50	>50	>50	>50	>50	>50	>50	>50	>50	>50	>50	>50	>50	>50	>50	>50
Vibrio campbellii	>50	>50	>50	>50	>50	>50	>50	>50	>50	>50	>50	>50	>50	>50	>50	>50	>50	42.06	>50	40.06	>50	>50	>50	>50	>50
Vibrio tubiashii	>50	>50	>50	>50	>50	>50	>50	>50	>50	>50	>50	>50	>50	>50	44.35	>50	>50	>50	>50	>50	>50	>50	>50	>50	>50
Vibrio hollisae	>50	>50	>50	>50	>50	>50	41.08	>50	>50	>50	>50	>50	>50	>50	>50	>50	>50	>50	>50	>50	>50	>50	>50	>50	>50
Vibrio parahaemolyticus	>50	>50	>50	>50	>50	>50	>50	>50	>50	>50	>50	>50	>50	>50	>50	>50	>50	>50	>50	>50	>50	>50	>50	>50	>50
Vibrio metschnikovii	>50	>50	>50	>50	>50	>50	>50	>50	>50	>50	>50	>50	>50	>50	>50	>50	>50	>50	>50	>50	>50	>50	>50	>50	>50
Vibrio mediterranei	>50	>50	>50	>50	>50	>50	>50	>50	>50	>50	>50	>50	>50	>50	>50	>50	>50	>50	>50	>50	>50	>50	>50	>50	>50
E. coli 10798	>50	>50	>50	>50	>50	>50	>50	>50	>50	>50	>50	>50	>50	>50	>50	>50	>50	>50	>50	>50	>50	>50	>50	>50	>50
Vibrio algosus	>50	>50	>50	>50	>50	>50	>50	>50	>50	>50	>50	>50	>50	>50	>50	>50	>50	>50	>50	>50	>50	>50	40.59	>50	47.17
Photobacterium leiognathi	>50	>50	>50	>50	>50	>50	>50	>50	>50	>50	>50	>50	>50	>50	>50	>50	>50	>50	>50	>50	>50	>50	>50	>50	43.16
Photobacterium angustum	>50	>50	>50	>50	>50	>50	>50	>50	>50	>50	>50	>50	>50	>50	>50	>50	>50	>50	>50	>50	>50	>50	>50	>50	>50
Vibrio abalonicus	>50	>50	>50	>50	>50	>50	>50	>50	>50	>50	>50	>50	>50	>50	>50	>50	>50	>50	>50	>50	>50	>50	>50	>50	42.4
Vibrio gazogenes	>50	>50	>50	>50	>50	>50	>50	>50	>50	>50	>50	>50	>50	>50	>50	>50	>50	>50	>50	>50	>50	>50	>50	>50	>50
Vibrio cincinnatiensis	>50	41.17	>50	>50	>50	>50	>50	>50	>50	>50	42.03	>50	>50	>50	>50	>50	>50	>50	>50	>50	>50	>50	>50	>50	>50
Aeromonas sobria	>50	>50	>50	>50	>50	>50	>50	>50	43.01	>50	43.4	>50	>50	>50	>50	>50	>50	>50	>50	>50	>50	>50	>50	>50	>50
Vibrio navarrensis	>50	>50	>50	>50	>50	>50	>50	>50	>50	>50	>50	>50	>50	>50	44.09	>50	>50	>50	>50	>50	>50	>50	>50	>50	18.68
Vibrio mytili	>50	>50	>50	>50	>50	>50	>50	>50	>50	>50	>50	>50	>50	>50	>50	>50	>50	>50	>50	>50	>50	>50	>50	>50	43.04
Vibrio aerogenes	>50	>50	>50	>50	>50	>50	>50	>50	>50	>50	>50	>50	>50	>50	>50	>50	>50	>50	>50	>50	>50	>50	>50	>50	45.06

LEGEND	O1	O139	non-O1/non-O139	non-O1		<20	21-24	25-28	29-32	33-36	37-39	>40	>50
